# Arterial Stiffness Response to Acute Combined Training with Different Volumes in Coronary Artery Disease and Heart Failure Patients

**DOI:** 10.3390/ijerph192214994

**Published:** 2022-11-14

**Authors:** Vanessa Santos, Luís Miguel Massuça, Vitor Angarten, Xavier Melo, Rita Pinto, Bo Fernhall, Helena Santa-Clara

**Affiliations:** 1 Exercise and Health Laboratory, CIPER, Faculdade de Motricidade Humana, Universidade de Lisboa, Cruz Quebrada, 1649-004 Lisboa, Portugal; 2 KinesioLab, Research Unit in Human Movement Analysis, Instituto Piaget, 2805-059 Almada, Portugal; 3 ICPOL Research Center, Higher Institute of Police Sciences and Internal Security, 1300-352 Lisbon, Portugal; 4 CIDEFES—Research Center in Sport, Physical Education, Exercise and Health, Lusófona University, 1749-024 Lisbon, Portugal; 5 Egas Moniz Interdisciplinary Research Center (CiiEM), Egas Moniz School of Health, 2829-511 Almada, Portugal; 6 Structural and Coronary Heart Disease Unit, Centro Cardiovascular da Universidade de Lisboa (CCUL@RISE), Faculty of Medicine, University of Lisbon, 1649-004 Lisboa, Portugal; 7 College of Nursing and Health Sciences, University of Massachusetts, 100 Morrissey Boulevard, Boston, MA 02125, USA

**Keywords:** arterial stiffness, coronary artery disease, heart failure, pulse wave velocity, combined exercise, endurance exercise, resistance exercise

## Abstract

Resistance training has been shown to acutely increase arterial stiffness (AS), while endurance training appears to decrease AS. However, the findings are from studies in apparently healthy subjects and have limited applicability to patients at low and high cardiovascular risk, for whom combined exercise is recommended. We compared the time course of changes in local and regional indices of AS in response to high-volume combined endurance training (CET) and high-volume combined resistance training (CRT) in patients with coronary artery disease (CAD) and heart failure (HF). We studied 20 men with CAD and HF (10 each) aged 68.3 ± 9.6 years. AS was measured by pulse wave velocity (PWV), and brachial and central blood pressure (BP) were determined after 15 min of rest and 5 and 15 min after the exercise session. All patients completed two sessions on nonconsecutive days. A protocol by time interaction effect was observed for carotid (η^2^ = 0.21, *p* = 0.02), aortic (η^2^ = 0.60, *p* < 0.001), and femoral (η^2^ = 0.46, *p* = 0.01) PWV after CET and CRT, suggesting that PWV decreased after CET and increased after CRT. Decreases in the brachial and central variables of BP across time points were observed in both protocols. CET decreased whereas CRT increased carotid, aortic, and femoral PWV at 15 min after exercise in patients with CAD and HF.

## 1. Introduction

Arterial stiffness (AS) is a hallmark of ageing and is closely related to many pathological conditions such as atherosclerosis, dyslipidemia, diabetes, and chronic kidney disease [1,2]. Increased AS has been observed in patients with coronary artery disease (CAD) and heart failure (HF) [3]. In patients with HF, increased AS is associated with cardiovascular morbidity and mortality [4]. Increased aortic stiffness is also associated with exercise intolerance in elderly patients with HF [5].

Aortic pulse wave velocity (PWV) is considered the “gold standard” for noninvasive measurement of AS. Aortic PWV has a strong and independent predictive value for cardiovascular and all-cause mortality even in patients with CAD, hypertension, or diabetes [6,7]. Each 1 m/s increase in PWV is associated with a 12–14% increased risk of cardiovascular events and a 13–15% increased risk of cardiovascular disease (CVD) mortality [8].

In apparently healthy subjects, a single bout of endurance training has been shown to increase AS [9,10,11,12,13], while acute endurance training has been shown to decrease AS [11,14,15,16,17,18]. The acute effects of resistance training appear to be strongly influenced by training intensity. High-intensity resistance training increases AS [9,19,20] although the effect lasts less than 60 min [9,21]. On the other hand, low-intensity resistance training appears to increase arterial compliance and decrease AS 30 and 60 min after exercise [20]. Overall, these results suggest different recovery responses for AS depending on the type and intensity of training [22]. For cardiac patients, the benefits of combined training instead of only one component (endurance or resistance) have already been demonstrated in the literature [23,24]. However, as far as we know, there are no studies in the literature on the acute effects of combined training in AS.

Much of the difference between endurance and resistance training can be attributed to the different endocrinological and molecular responses between the two types of training (endurance and resistance) [25]. It seems that the aortic PWV also responds differently to these two types of training [26]. Therefore, it is important to understand the acute responses that combined training loads may elicit, as acute responses are known to cause chronic adaptations [27].

Thus, the aim of this study was to compare the time course of changes in local and regional indices of AS after exercise training in response to two combined exercise training protocols in patients with CAD and HF.

## 2. Materials and Methods

### 2.1. Participants

Thirty-two male cardiac patients were recruited to participate in the study. The inclusion criteria were male, over 18 years of age, and diagnosis of stable CAD or HF. HF had to have a left ventricular ejection fraction (LVEF) < 45%, and patients with CAD had to have a preserved ejection fraction (LVEF stablished > 50%) and exercise regularly for at least one month. In addition, participants were excluded from the study if they had diabetes, musculoskeletal problems, or a body mass index (BMI) > of 35 kg/m^2^. Nineteen of the participants had CAD and 13 had HF, all of whom had been participating in a sports rehabilitation program for at least one month. Of these, one patient with CAD and one patient with HF were excluded for medical reasons, three patients with CAD refused to participate, five patients with CAD did not complete both sessions, and two patients with HF did not meet the inclusion criteria. A total of 20 patients (10 with CAD aged 73.2 ± 10.3 years and 10 with HF aged 63.4 ± 6.2 years) had complete data for both training sessions.

All subjects gave their informed consent in writing prior to participation. The Ethics Committee of the Faculty of Human Kinetics of the College of Lisbon approved this study (approval number: N28/2017), and all procedures and the treatment of subjects were in accordance with the Declaration of Helsinki.

### 2.2. Study Design

This was a cross-over exercise protocol with repeated measures. Participants participated in two separate interventions consisting of one session of combined exercise with a higher volume of endurance training (CET) and one session of combined exercise with a higher volume of resistance training (CRT), at least 48 h apart. Anthropometric measurements including weight, height, and BMI were obtained the week before. Participants had participated in these types of training sessions for at least 1 month. Participants were assessed at rest (15 min supine in a quiet, dimly lit, temperature-controlled laboratory) and 5 and 15 min after the exercise intervention. These time points were selected based on previous results [27,28,29]. Heart rate was measured continuously throughout the exercise session using a Polar band (H10 Polar, Electro, Kempele, Finland), and rated perceived exertion (RPE) was assessed [30].

All patients performed a maximal cardiopulmonary exercise test according to the Bruce protocol and an assessment of the maximum at one repetition (RM) on all devices during the week prior to examinations. 

All sessions were conducted in the morning, with each participant completing each session at the same time of day to minimize potential diurnal variation. Participants were instructed not to consume any food or drink other than water after midnight before the sessions and to abstain from alcohol, caffeine, and exercise for at least 24 h before each session.

### 2.3. Intervention Sessions

CET and CRT interventions were designed to represent typical sessions according to current guidelines for patients with CVD [22,31,32]. In both sessions, strength training was performed before endurance training, and the duration of each session was 45 min. CRT consisted of a set of eight repetitions of resistance exercises on six weight machines (three for the upper limbs and three for the lower limbs) performed at 70% of 1 RM, with 30 s rest between exercises. This was followed by supervised treadmill training using high-intensity interval training with 10 interval training periods (2 min at 85–90% of maximum heart rate) and 9 rest periods (1 min passive rest) between sets. The CRT consisted of three sets of eight repetitions of resistance-based exercises with the same six weight machines at 70% of 1 RM, with 30 s rest between exercises and 1 min rest between sets. This was followed by supervised treadmill training using the same high-intensity interval training as described above but with only five interval training periods (2 min at 85–90% of maximum heart rate) and four rests (1 min passive rest) between interval training periods. Both training sessions consisted of a 5 min warm-up program that included two upper-limb exercises and two lower-limb exercises repeated 12 times at 30–40% 1 RM. The method of endurance training was chosen because there is growing evidence that high-intensity interval training is more effective than continuous moderate-intensity training in improving cardiorespiratory fitness in cardiac patients [33,34].

Heart rate was continuously monitored, and exercise physiologists repeatedly insisted on avoiding the Valsalva maneuver.

### 2.4. Central and Peripheral Arterial Stiffness Indices

Blood pressure (BP) was measured twice on the right arm with an automatic cuff (OMROM 907, Tokyo, Japan). If a difference of more than 10 mmHg was detected on the systolic BP between the two measurements, another BP was performed. We calculated the mean arterial pressure (MAP) and pulse pressure (PP) for adjustment purposes using the following equations: MAP = diastolic BP + 1/3 (systolic BP—diastolic BP); PP = systolic BP—diastolic BP.

All measurements were performed in the supine position after the patient had rested for 15 min before the session and again at 5 and 15 min after each exercise session. An ultrasound machine with a 13 MHz linear probe (MyLab One, Esaote, Italy) with Quality Arterial Stiffness technology was used, based on a high-frequency signal in the right carotid segment ~1 cm before the bifurcation. The right carotid pressure curve was calibrated to the right brachial artery diastolic pressure curve by iteratively changing the wall stiffness coefficient and MAP. Carotid PWV was calculated with the following equation: $\mathrm{PWV}=\frac{1}{\sqrt{\rho \;\cdot \;\mathrm{DC}}}=\sqrt{\frac{D^{2}\;\cdot \;\Delta \mathrm{PP}}{\rho \;\cdot \;\left(2\;\cdot \;D\;\cdot \;\Delta D\;+\;{\Delta D}^{2}\right)}}$, where D: diastolic diameter; ΔD: change in diameter in systole; DC: distensibility coefficient; ΔPP: local pulse pressure; ρ: blood density [35]. Regional PWV was also measured by applanation tonometry immediately after ultrasound. The carotid, femoral, and distal posterior tibial arteries on the right side of the body were located by a single operator, and the point for acquisition of the corresponding pressure curves was marked with two specific pressure-sensitive transducers. The distance between the carotid, femoral, radial, and distal posterior tibial arteries was measured directly and entered into Complior analysis software using a correction factor of 0.8 (ALAM Medical, Paris, France) (ALAM Medical, Paris, France). The right brachial BP was measured and entered into the software, and then, signal acquisition was started. Whenever a continuous drop before the sharp systolic upstroke was not clearly seen, or the tolerance was above 0.5 m/s, a third second measurement was taken.

### 2.5. Statistical Analysis

The power and sample-size calculations (G-Power, version 3.1.3) were based on a predicted central PWV difference of 0.9 m/s with an SD of 0.1 m/s [36], α = 0.05, 1 − β = 0.80 and an expected dropout rate of 20%. According to these calculations, nine patients were required in each group. Comparisons between protocols (CET vs. CRT) over time (rest vs. post-intervention time points) for AS indices were examined using three-way (protocols × time) repeated-measures analysis of variance (ANOVA) adjusted for crossover analysis. Bonferroni tests were used for post hoc comparison of means between each pair of groups. Because AS depends on arterial pressure [37], the percent change in MAP from rest to 15 min after exercise was included as a covariate.

## 3. Results

The characteristics of the patients with CAD (*n* = 10) and HF (*n* = 10) who participated in the study are shown in Table 1. All participants achieved 85–90% of maximal heart rate during both sessions. RPE values ranged from 7–9 in both sessions, indicating high effort (Table 2).

**Figure 1:**
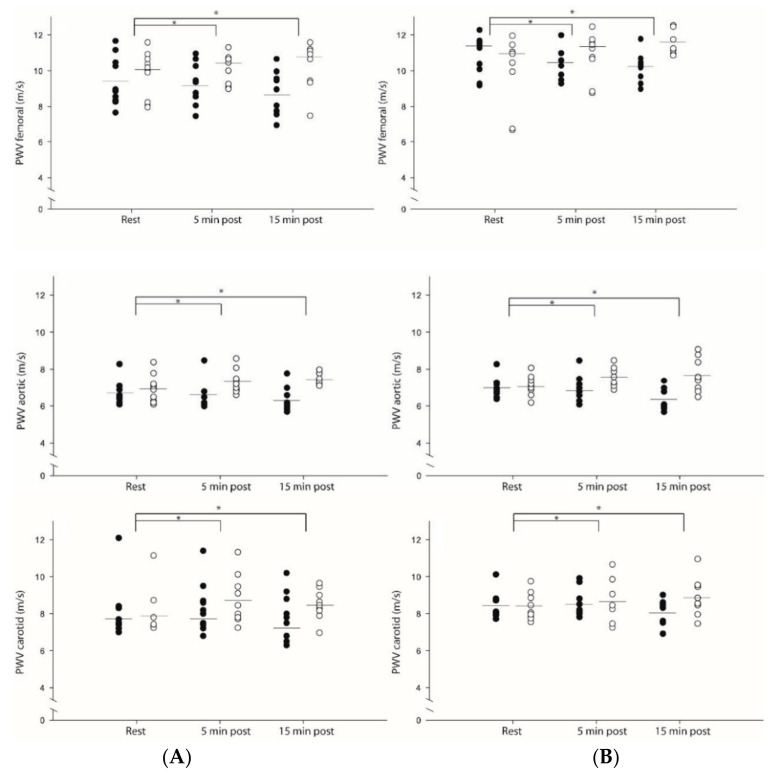
Carotid, aortic, and femoral pulse wave velocity (PWV) at rest and throughout the post-exercise period on (**A**) coronary artery disease (CAD) patients and (**B**) heart failure (HF) patients. Data are presented as (⬤) CET—endurance training, and (⚪) CRT—resistance training. *, significant difference from baseline within group: *p* < 0.05.

A protocol by time interaction effect was observed for carotid (η^2^ = 0.21, *p* = 0.02), aortic (η^2^ = 0.60, *p* < 0.001), and femoral PWV (η^2^ = 0.46, *p* = 0.01) after CET and CRT, with no differences between patients with CAD and HF, suggesting that PWV decreased after CET (CAD: −0.31 m/s, −0.43 m/s, and −0.77 m/s, respectively; HF: −0.4 m/s, −0.62 m/s, and −0.8 m/s, respectively) but increased after CRT (CAD: +0.62 m/s, +0.53 m/s, and +0.45 m/s, respectively; HF: +0.44 m/s, +0.6 m/s, and +1.44 m/s, respectively; Figure 1).

Table 3 shows the significant main effects of brachial and central BP between sessions and pathologies. Significant main effects in time were found for CAD and HF patients in brachial and central BP variables from rest in both moments (decreases in all time points, with *p* < 0.05), but there were no interaction effects between the group (CAD and HF) and the protocol (CET and CRT) (Table 3), suggesting a decrease over time regardless of the session or the pathology. 

## 4. Discussion

To our knowledge, this was the first study to compare changes in local and regional indices of AS after combined endurance and resistance training in cardiac patients diagnosed with CAD or HF. AS decreased after CET but increased after CRT in both CAD and HF. These results suggest that CET is associated with an acute reduction in cardiovascular risk despite equal exercise, whereas CRT may be associated with periods of increased risk caused by transient changes in AS immediately after exercise. However, it is unclear whether these transient changes in AS actually increase or decrease risk. For example, angina occurs more frequently in cardiac patients after endurance exercise than after resistance exercise [39]. This discrepancy is probably due to the potential increase in cardiac perfusion during acute resistance training, which is dependent on increased DBP [40]. However, one of the most important findings of the present study is that although both protocols included resistance exercise, AS still decreased in the protocol with the lowest volume of resistance exercise.

Previous studies have supported the notion that aerobic or resistance exercise training lowers systolic aortic pressure because of improvements in vasoactive agents and endothelial function [41,42,43]. On the contrary, Heffernan et al. (2013) found that the reduction in central BP in response to resistance training is due to the reduction in reservoir pressure, which is proportional to the volume of blood stored in the aorta and, in turn, depends on the interactions between systemic arterial compliance and impedance of the exit tract [44,45]. However, Figueroa et al. (2014) and Taaffe et al. (2007) reported that this can be attributed to improved peripheral muscular arterial dilation and peripheral vascular resistance [41,43].

To our knowledge, there are no studies on the acute effects of combined exercise training on AS in individuals with or without CVD. However, there are several studies in the literature with healthy individuals in which acute endurance training reduced AS [10,11,14,18] and also in patients with CAD; Sung et al. (2009) and Trachsel et al. (2019) showed the same results, and most acute effects of resistance training showed an increase in AS only in healthy subjects [9,11,13]. Regarding the protocols tested in the present study, the protocol with the highest volume of endurance decreased the AS, while the protocol with the highest volume of resistance exercise increased the AS, which is consistent with the results when the endurance and resistance components are examined separately.

Exercise training can induce changes similar to those observed following the protocol with the highest volume of endurance exercise. Zhang et al. [46], in a meta-analysis of exercise training studies lasting at least four weeks, showed that combined training significantly improved aortic PWV in patients with CVD. The intensity of the resistance protocol was similar to that of our high-resistance-volume protocol. Li et al. (2015) [47] studied normotensive and hypertensive adults and concluded that combined training may have beneficial effects on AS when endurance and resistance training occurs in the same training session. In contrast to previous meta-analyses, Ashor et al. [8] found no significant differences in aortic PWV with combined training. Montero et al. [48] also showed decreased aortic PWV in the training groups, which reached statistical significance in the endurance but not in the combined training groups compared with controls. This particular meta-analysis suggests that combined endurance and resistance training, although leading to a nonsignificant reduction in PWV, has less impact on AS than aerobic training alone. However, combined training can be recommended due to its beneficial effects on body composition and musculoskeletal health as well as its metabolic benefits compared to aerobic training alone [48]. Chrysohoou et al. (2015) [49] studied patients with chronic HF and showed that PWV did not improve in response to combined training.

Endothelium-dependent vasodilation is impaired in HF, which may influence our results. We know from the literature that the pathophysiological mechanism of endothelial dysfunction in patients with HF is related to increased oxidative stress, which exacerbates pre-existing vasoconstriction and increases myocardial damage. Decreased coronary endothelium-dependent vasodilation impairs myocardial perfusion, reduces coronary flow, and worsens ventricular function [50]. Reduction in nitric oxide synthesis (NO), involvement of a genetic polymorphism for endothelial NO (eNOS) synthase, oxidative inactivation of NO, and increased levels of asymmetric dimethylarginine, an endogenous eNOS inhibitor, increased levels of oxidized low-density lipoprotein in plasma, increased endothelium-bound xanthine oxidase activity, and decreased endothelium-bound extracellular superoxide dismutase activity. MicroRNAs are downregulated in chronic HF endothelial dysfunction, and this feature may have implications for endothelial dysfunction in this patient population [51]. Hambrecht et al. (1998) showed that improvement in endothelial function was associated with an increase in exercise capacity in patients with HF [52]. One of the main characteristics of these patients was muscle weakness and low muscle mass, which contributed to poor physical performance. Regular exercise improves muscle function and physical performance. It also improves the ability of blood vessels to dilate in response to exercise, diastolic function of the left ventricle, and neurohormonal activation, which improves endothelial nitric oxide formation and consequent vasodilation of vessels of the skeletal muscle [53]. Endothelial dysfunction is also associated with decreased arterial distensibility [54].

Our results show that in low- and high-risk patients, such as CAD and HF, CET decreases AS, and CRT increases arterial stiffness. A higher volume of endurance training helps to reverse the effect induced by resistance training.

This study is not without limitations. The sample studied consisted only of men because the rehabilitation programs in which recruitment took place are predominantly attended by men, which does not allow for a gender-homogeneous sample (94% are men). The order of experimental interventions was not communicated to participants in a blinded fashion because of limited options. Post-exercise monitoring of AS responses at 5 and 15 min may have resulted in failure to detect subtle changes or those outside this period. Previous studies have found sex differences in both resting aortic PWV and after acute endurance exercise [55]. However, the selection of an exclusively male cohort allowed us to assess the typical physiological responses to acute exercise in this particular population without the influence of sex.

## 5. Conclusions

Different combined exercise protocols have different effects on central and peripheral AS in patients with CAD and HF. CET decreases cardiovascular, aortic, and femoral PWV, and CRT increases cardiovascular, aortic, and femoral PWV after a 15 min session in patients with CAD and HF. However, further studies are needed to understand the different volumes in this type of combined exercise.

In conclusion, we note that these results have important implications for the rehabilitation of these patients not only for the patients themselves but also for health professionals, as they allow tailoring individual treatments to specific cardiovascular pathologies.

## Figures and Tables

**Table 1:** Characteristics of the patients.

	CAD	HF
Age (years)	73.2 ± 10.0	63.4 ± 6.1
Weight (kg)	73.8 ± 9.2	81.1 ± 17.0
Height (m^2^)	1.7 ± 0.1	1.7 ± 0.1
BMI (kg/m^2^)	26.6 ± 3.1	27.7 ± 4.4
Brachial SBP at rest (mmHg)	119.3 ± 10.9	123.2 ± 7.6
Brachial DBP at rest (mmHg)	67.8 ± 8.1	69.7 ± 6.3
Hypertension (%)	60	50
Hyperlipidemia (%)	80	80
Overweight/obesity (%)	20	10
>1-year Ex-smoker (%)	0	10
>1-year Ex-depression (%)	20	40
Beta-blocker (%)	100	100
ACEi/ARB (%)	80	100
Statin (%)	80	100
Antiplatelet (%)	100	50
Diuretics (%)	60	60
CCB (%)	40	10
LVEF (%)	>50	30.6 ± 5.9

ACEi/ARB, angiotensin-converting enzyme inhibitor/angiotensin receptor blocker; BMI, body mass index; CAD, coronary artery disease; CCB, calcium channel blocker; DBP, diastolic blood pressure; HF, heart failure; LVEF, left ventricular ejection fraction; SBP, systolic blood pressure.

**Table 2:** Heart rate session variables.

	CAD		HF	
	CET	CRT	CET	CRT
Heart rate rest (bpm)	63 ± 7	67 ± 8	67 ± 8	65 ± 6
Peak heart rate (bpm)	119 ± 6	117 ± 8 †	111 ± 8	109 ± 5 †
TRIMP	96.1 ± 12.6	82.4 ± 8.0	96.3 ± 12.7	91.3 ± 12.1
RPE	8 ± 1	8 ± 1	8 ± 1	8 ± 1
Heart rate recovery 5 min (bpm)	94 ± 20 *	99 ± 16 *†	99 ± 21 *	97 ± 14 *†
Heart rate recovery 15 min (bpm)	88 ± 18 *	91 ± 12 *†	92 ± 16 *	89 ± 12 *†

†, *p* < 0.05 significantly different between groups; *, *p* < 0.01 significantly different from rest; bpm, beats per minute; CAD, coronary artery disease; CET, higher volume of endurance training; CRT, higher volume of resistance training; HF, heart failure; RPE, rated perceived exertion; TRIMP, training impulses, according to the method by Edwards [38].

**Table 3:** Arterial stiffness indices at rest and after combined exercise sessions in both pathologies.

Variables	Time Point	Mean ± SD CAD	Mean ± SD HF	Main Effect of the Protocol for CAD	Main Effect of Time for CAD	Main Effect of the Protocol for HF	Main Effect of Time for HF
bSBP	Rest	119.30 ± 10.67	123.15 ± 7.57	0.05 (*p* = 0.95)	4.39 (*p* = 0.03) *	0.72 (*p* = 0.50)	8.25 (*p* < 0.01) *
	5 min post	118.55 ± 9.83	121.75 ± 11.14				
	15 min post	115.70 ± 10.33	120.10 ± 8.36				
bDBP	Rest	69.75 ± 8.07	74.70 ± 6.34	0.37 (*p* = 0.70)	3.68 (*p* = 0.05) *	0.39 (*p* = 0.68)	11.06 (*p* < 0.01) *
	5 min post	67.70 ± 5.45	71.00 ± 8.54				
	15 min post	66.80 ± 8.13	69.30 ± 6.27				
MAP	Rest	86.82 ± 8.38	93.46 ± 5.93	0.38 (*p* = 0.69)	6.06 (*p* = 0.01) *	0.58 (*p* = 0.57)	8.39 (*p* < 0.01) *
	5 min post	84.00 ± 6.23	90.18 ± 8.92				
	15 min post	83.10 ± 8.15	86.21 ± 6.13				
bPP	Rest	54.15 ± 7.31	58.25 ± 3.67	0.14 (*p* = 0.87)	6.24 (*p* = 0.01) *	1.35 (*p* = 0.29)	7.25 (*p* = 0.01) *
	5 min post	51.15 ± 7.49	56.45 ± 5.05				
	15 min post	50.15 ± 8.47	52.30 ± 6.39				
aSBP	Rest	114.30 ± 13.88	129.25 ± 7.70	0.11 (*p* = 0.90)	4.69 (*p* = 0.02) *	0.47 (*p* = 0.64)	7.35 (*p* = 0.01) *
	5 min post	112.25 ± 11.55	121.95 ± 10.68				
	15 min post	109.55 ± 13.02	118.95 ± 7.93				
aDBP	Rest	69.80 ± 8.08	74.75 ± 6.43	0.39 (*p* = 0.68)	3.57 (*p* = 0.05) *	0.41 (*p* = 0.67)	11.21 (*p* < 0.01) *
	5 min post	67.70 ± 5.45	70.00 ± 8.54				
	15 min post	66.90 ± 8.41	69.30 ± 6.27				
aPP	Rest	50.60 ± 9.38	57.55 ± 5.03	0.03 (*p* = 0.97)	4.69 (*p* = 0.02) *	1.15 (*p* = 0.34)	4.97 (*p* = 0.02) *
	5 min post	47.15 ± 9.60	55.50 ± 4.42				
	15 min post	46.40 ± 9.7	51.90 ± 6.97				

*, *p* < 0.05 significantly different; aDBP, aortic diastolic blood pressure; aSBP, aortic systolic blood pressure; bDBP, brachial diastolic blood pressure; bPP, brachial pulse pressure; bSBP, brachial systolic blood pressure; CAD, coronary artery disease; HF, heart failure; MAP, mean arterial pressure; aPP, aortic pulse pressure.

## Data Availability

All data relevant to the study are included in the article.
